# Complete genome sequence of *Lacticaseibacillus paracasei* strain VHProbi M56, isolated from yogurt

**DOI:** 10.1128/MRA.00537-23

**Published:** 2023-10-17

**Authors:** Chaoqun Guo, Hongchang Cui, Qian Wang, Zhi Duan

**Affiliations:** 1 Qingdao Vlandbiotech Group Co. Ltd., Qingdao, China; University of Maryland School of Medicine, Baltimore, Maryland, USA

**Keywords:** complete genome sequence, *Lacticaseibacillus paracasei*, VHProbi M56

## Abstract

*Lacticaseibacillus paracasei* strain VHProbi M56 is a probiotic strain that was isolated from yogurt. Here, we report the whole-genome sequence of this bacterium. The whole genome contains a chromosome and a plasmid.

## ANNOUNCEMENT

An amount of 10 g of yogurt collected from herdsman family in Tibet (91.1°E, 29.6°N) was serially diluted in the de Man-Rogosa-Sharpe (MRS) broth medium. Then, the 10^−7^ dilution was spread onto the MRS agar plate and incubated at 37°C for 36 h under anaerobic conditions to enrich bacteria. A colony designated VHProbi M56 was picked up and identified as the species *Lacticaseibacillus paracasei* using 16S rRNA sequencing, as described by Liu et al. (1). A combined strategy with both Illumina HiSeq 2500 and the PacBio RS II single molecule real-time (SMRT) platform was used to sequence the genome of this strain. The strain was cultured in the same growth condition as isolation for DNA extraction. Total DNA was extracted using the Genomic DNA Purification Kit (Promega, USA) following its instruction and used both for the Illumina and the PacBio library preparation. Template DNA was sheared into 400-bp fragments for the Illumina library creation using the TruSeq Library Preparation Kit from Illumina. The sheared fragments were used to prepare the Illumina libraries using the NEXTflex Rapid DNA-Seq Kit (Bio Scientific, USA). The prepared libraries were then used for paired-end Illumina sequencing (2 × 150 bp). For the PacBio sequencing, DNA was spun in a Covaris G-Tube (Covaris, MA) to be sheared into fragments. Then, ~10 kb fragments were purified, end repaired, and ligated with the SMRTbell sequencing adapters following the PacBio recommendation. Next, an ~10-kb insert library was prepared using SMRTbell Express Template Prep Kit 2.0 and sequenced on one SMRT cell using standard methods. Finally, 8,445,782 raw reads and 8,366,374 clean reads were generated using the Illumina sequencing platform to reach a depth of 1107.22-fold coverage, and 364,255 raw reads (*N*_50_, 230,420 bp) were generated using the PacBio sequencing platform. For Illumina raw data, adapters, non-A, G, C, T bases of the 5′ end, reads containing >10% unknown *N,* and low-quality reads were removed by using Sickle v1.33 (https://github.com/najoshi/sickle). Clean data were assembled into a scaffold using SOAPdenovo v2.04 (2). The PacBio raw reads were then assembled into a scaffold using hierarchical genome assembly process (HGAP4.0) (3) and Canuv2.2 (4). Error correction of the PacBio assembly results was performed by Pilonv1.22 (5) using the Illumina reads. Since there was a 5,000-bp overlap of the two ends, then one end of the overlap was cut off to circularize the sequence. The final assembly generated a complete genome with a seamless chromosome and a plasmid. Glimmer v3.02 (6), tRNA-scan-SE v2.0 (7), and barrnap v0.9 were used to predict protein-coding genes, tRNA, and rRNA, respectively (https://github.com/tseemann/barrnap/).CheckM v1.1.3 was used to check the quality of the assembled genome sequence with the completeness of 99.18%, contamination of 0%, and strain heterogeneity of 0% (8). Genome annotations were conducted using the NCBI Prokaryotic Genome Annotation Pipeline (PGAP) v5.3 (9). Default parameters were used for all software unless otherwise specified.

The whole-genome sequence of *L. paracasei* VHProbi M56 contains a 3,039,086-bp circular chromosome and a 43,381-bp circular plasmid with GC contents of 46.40%. We stated that the additional molecule is a plasmid verified by Pilon v1.22.There were 2,870 protein-coding genes, 15 rRNA genes (5S rRNA, 16S rRNA, and 23S rRNA), 61 tRNA genes, 3 ncRNA genes, and 50 pseudogenes in the genome.

## Data Availability

The 16S rRNA sequence and complete genome sequence of *L. paracasei* VHProbi M56 have been deposited in the database with GenBank accession numbers OQ592688 and CP094959 to CP094960, SRA accession numbers SRR21227525 and SRR21227526, BioProject accession number PRJNA821984, BioSample accession number SAMN27162336.
